# Antigen-Specific Tissue-Resident Memory T Cells in the Respiratory System Were Generated following Intranasal Vaccination of Mice with BCG

**DOI:** 10.1155/2021/6660379

**Published:** 2021-03-27

**Authors:** Qiongli Wu, Shuangpeng Kang, Jun Huang, Shunqiao Wan, Binyan Yang, Changyou Wu

**Affiliations:** ^1^Institute of Immunology, Zhongshan School of Medicine, Sun Yat-sen University, Guangzhou 510080, China; ^2^Key Laboratory of Immunology, Sino-French Hoffmann Institute, Guangzhou Medical University, Guangzhou, China

## Abstract

Tissue-resident memory T cells (T_RM_) are different from effector memory T cells (T_EM_) and central memory T cells (T_CM_) and contribute to the protective immunity against local challenges. Currently, we found that CD4^+^ and CD8^+^ T_RM_ cells in the nasal mucosa, trachea, lungs, and lavage fluids were heterogeneous on the expression of CD69 and CD103 as well as the production of cytokines including IFN-*γ*, IL-2, and TNF-*α*. After intranasal vaccination of mice with BCG, respiratory tissues expressed higher levels of the chemokine CXCL16 and T_RM_ cells expressed CXCR6 to CXCL16. In addition, antigen-specific CD4^+^ and CD8^+^ T_RM_ cells expressed cytokines following the stimulation with BCG and persisted in the nasal mucosa, trachea, and lungs for more than a hundred days. At the same time, mice were infected intranasally with live BCG and the results showed that vaccinated mice cleared up live BCG faster than nonvaccinated mice in the respiratory system. Taken together, our data demonstrated that intranasal vaccination of mice with BCG could induce antigen-specific CD4^+^ and CD8^+^ T_RM_ cells in the respiratory system and have the ability to provide protection against pulmonary reinfection.

## 1. Introduction

Recent studies have demonstrated that both circulating memory T cells and tissue-resident memory T cells (T_RM_) abundant in peripheral tissues play a central role to elicit protective immunity [1, 2]. Different from effector memory T cells (T_EM_) and central memory T cells (T_CM_), T_RM_ cells cannot recirculate through the blood and lymphatic system [3]. On the contrary, they reside within certain nonlymphoid tissues (NLT) including the skin, mucous membranes, urogenital system, brain, lung, and liver where they make critical contributions to protective immunity against local challenge [4–8]. It has been reported that the main source of T_RM_ is the induction of BATF3^+^ DC to induce the differentiation of naive T cells (T_N_) and T_CM_ into precursor T_RM_ cells (pT_RM_) and enter the tissues where T_RM_ expresses CD69 under the role of IFN-*α*. Afterwards, T_RM_ migrates to the epithelial tissues and expresses CD103 under the action of TGF-*β*. Besides, effector T cells (T_EFF_) enters the epithelial tissues to recognize antigens and the transcription factor KLF2 is reduced instantaneously, leading to the downregulation of S1PR1 and the upregulation of CD69. At the same time, TNF, IFN-*α*, and IL-33 induced the cells to express CD103 and reside in the tissues [9]. T_RM_ cells can not only play the defense response directly and quickly when the pathogens invade in the tissues but also promote tissue repair and maintain tissue immune homeostasis. In addition, T_RM_ cells also play a very important role in the immune responses to tumors, allergies, and autoimmune diseases. As early as 2001, CD8^+^ T_RM_ cells were discovered and subsequently demonstrated to play a cytotoxic role in lung, skin, vagina, and respiratory tissues [4]. In 2012, Teijaro et al. confirmed that CD4^+^ T_RM_ cells also exist in lung tissues to protect mice against influenza virus infection [10].

It is worth noting that the respiratory system is a site of many common diseases and innate as well as adaptive immunity plays an extremely important role in the prevention and control of the diseases [11]. Respiratory diseases accounted for the fourth leading (13.1%) cause of death in cities and third in rural areas (16.4%) in China. Tuberculosis remains the most important cause of respiratory diseases worldwide. However, the licensed TB vaccine BCG does not provide effective protection for all age groups, particularly in adults [12]. Therefore, effective vaccines, optimized vaccination routes, and targeted activation of immune cells are the keys to strengthen immunity. T_RM_ cells are resident in mucous membranes and induced by vaccination to provide rapid and effective protective immunity against infection and are the basis for successful vaccination [9, 13]. The mucous membranes of the respiratory system include the nasal mucosae of the upper respiratory system and the trachea and lungs of the lower respiratory system. T_RM_ cells of respiratory system can be activated in situ by intranasal vaccination, which has a very good immune effect [14, 15]. T_RM_ cells not only play a direct role but also activate the innate immune responses against reinfection [16]. More and more studies suggested that T_RM_ cells can play the defense response directly and quickly when the pathogens invade in the tissues [17].

Recently, a new type of coronavirus was identified and named 2019 novel coronavirus (COVID-19) by the World Health Organization (WHO) [18]. Arina et al. reported that T_RM_ cells are far superior to circulating memory T cells in reducing viral load upon rechallenge even in the absence of new infiltrating T cells [19]. This suggests that T_RM_ cells will play an important role in preventing COVID-19 in the future.

However, the phenotype, function, and regulation characteristics of T_RM_ cells in the respiratory system are less studied. To answer this, we established the murine model of BCG immunization to compare the difference in phenotype and function of T_RM_ cells in lavage fluid, nasal mucosa, trachea, and lung tissues and explored the similarities and differences in function and regulation. The results indicated that the BCG could induce the expression of IFN-*γ*, TNF-*α*, and IL-2 by T_RM_ cells in lavage fluids, nasal mucosa, trachea, and lung tissues. Besides, T_RM_ cells generated by BCG could protect the infection of live BCG. Meanwhile, T_RM_ cells were increased in the respiratory system after intranasal vaccination of mice with BCG. BCG-specific T_RM_ cells were persisted for a long time in lavage fluids, nasal mucosa, trachea, and lung tissues and had the ability to provide protection against pulmonary reinfection.

The study will reveal the immunology characteristics of T_RM_ cells in the respiratory system and provide a new theoretical basis for the prevention and treatment of respiratory infections and allergic diseases.

## 2. Materials and Methods

### 2.1. Animals

Female C57BL/6 mice aged 6–8 weeks were purchased from the Laboratory Animal Center of Sun Yat-sen University (S.C. XK 2016-0029) and housed in a specific pathogen-free condition at Sun Yat-sen University. The age and weight matching of mice was applied for all mouse-related experiments. All of the animal studies were approved by the Zhongshan School of Experimental Animal Ethics Committee, Sun Yat-sen University, Guangzhou, China.

### 2.2. Antigen and Immunization

**Figure 1:**
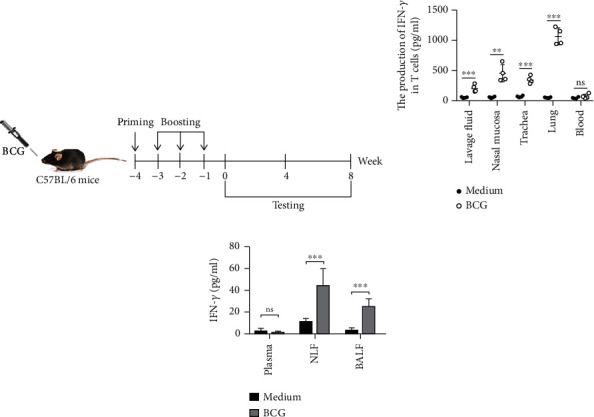
Vaccination of mice with BCG induced the production and expression of IFN-*γ* by T cells in the lavage fluids, nasal mucosa, trachea, and lungs. (a) Scheme of immunization. (b) Respiratory system tissues of lavage fluids nasal mucosa, trachea, lung, and blood cells were stimulated for 48 hrs with or without BCG plus anti-CD28 in the plate. The culture supernatants were detected for the production of INF-*γ* by ELISA. (c) The levels of IFN-*γ* in plasma, BALF, and NLF were detected by ELISA as mean ± SEM. Statistical significance was determined with two-way ANOVA. ^∗∗^*P* < 0.01 and ^∗∗∗^*P* < 0.001; ns: no significance.

Bacille calmette-guerin (BCG) was purchased from Chengdu Institute of Biological Products (Chengdu, China). The mice were immunized intranasally with the BCG (1 × 10^6^ CFU/mouse) in a volume of 20 *μ*l/mouse in PBS after anesthesia with isoflurane. The details of priming and boosting vaccination approach would be seen in Figure 1(a).

### 2.3. Reagents and Antibodies

Purified anti-CD3 (clone 145-2C11) and anti-CD28 (clone CD28.2) mAbs were purchased from BD Biosciences (San Jose, CA, USA). Phorbol myristate acetate and ionomycin were purchased from Sigma-Aldrich (St. Louis, MO, USA). Zombie Green™ Fixable Viability Kits were purchased from BioLegend (San Diego, CA). The antibodies that are used for cell surface staining and intracellular staining are listed in Table 1.

### 2.4. In Vivo Staining

To distinguish cell residence in tissues and circulation in blood, we label circulatory cells by CD45 antibodies intravenously (i.v.). In short, anti-CD45 phycoerythrin (clone 30-F11) was diluted with 10 *μ*g/ml in sterile PBS and was injected i.v with 200 *μ*l of the solution via the tail vein 5 min before sacrificing the mice.

### 2.5. Sample Collection and Cell Preparation

Control and vaccinated C57BL/6 mice were sacrificed at the indicated time points. Samples were collected from five to nine mice per group. Blood was obtained by eye socket bleeding and the serum was stored in −80°C refrigerator. The total cells of blood were suspended and isolated by Ficoll-Hypaque (Tianjin HaoYang Biological Manufacture, Tianjin, China) density gradient centrifugation according to the protocol. The bronchoalveolar lavage fluids (BALF) were acquired by lavaging the lungs with 250 *μ*l complete RPMI 1640 medium four times. The cells in the BALF were collected by centrifugation. The lungs, trachea, and nasal mucosa tissues were cut into 1–2 mm pieces and transferred to culture dishes containing 5 ml RPMI 1640 medium containing 2 mg/ml collagenase I and 1 mg/ml DNase I (Sigma-Aldrich, St. Louis, MO), followed by 2 hr incubation at 37°C in a shaker. The samples from the lung, spleen, trachea, and nasal mucosa tissues were mechanically disrupted and filtered through a 40 *μ*m cell strainer (Falcon, Durham, NC, BD) and isolated by percoll gradient (GE Healthcare) and washed twice with complete RPMI 1640 medium. Ultimately, the cells were counted and suspended in complete RPMI 1640 medium at a final concentration of 2 × 10^6^ cells/ml.

### 2.6. ELISA Assay for Cytokines

The cells (2 × 10^6^ cells/ml) were suspended in complete RPMI 1640 medium and stimulated for 48 hrs with or without BCG (50 *μ*g/ml) in the presence of anti-CD28 and anti-CD3 as positive control in round-bottomed 96-well plate, 200 *μ*l/well, at 37°C and 5%CO_2_. The culture supernatants were harvested for the detection of mouse IFN-*γ*. The sensitivity of the ELISA kit is 3.1 pg/ml for IFN-*γ*. All of the ELISA assays were performed according to the manufacturer's instructions. In brief, microwells were coated with capture antibody and incubated overnight at 4°C. The plates were washed with 0.05% Tween 20/PBS and blocked with 1% bovine serum albumin (BSA) in PBS at room temperature for 1 hr. Serum samples and serial dilution standards were added into the plates and incubated at 37°C for 2 hrs. The detection antibodies were added and incubated. After being washed with 0.05% Tween 20/PBS and developed by TMB substrate, the reaction was stopped with 10% H_2_SO_4_ and the OD value was assessed by a microplate wavelength of 450 nm. The actual cytokine concentration was calculated via the equation deducted from standards.

### 2.7. Flow Cytometry Analysis

The detection of surface markers and intracellular cytokines was performed as described below. In short, for cell surface marker staining, the cells were washed with PBS buffer containing 0.1% BSA and 0.05% sodium azide. For intracellular cytokine staining, the cells (2 × 10^6^ cells/ml) were stimulated for 6 hrs with PMA plus ionomycin at 37°C and 5% CO_2_ in the presence of brefeldin A (10 *μ*g/ml, Sigma-Aldrich, USA). The cells were washed twice with PBS and labeled with surface markers and dead/live streaming antibodies for 30 min at 4°C in dark. Then, the cells were washed twice with PBS buffer containing 0.1% BSA and 0.05% sodium azide and fixed with 4% paraformaldehyde, followed by permeabilizing in PBS buffer (0.1% BSA, 0.1% saponin, and 0.05% sodium azide) overnight at 4°C. All of the stained cells were assayed by FACSAria II (Becton Dickinson, San Jose, USA), and the data were analyzed by FlowJo software (Treestar, San Carlos, USA). The antibodies used are shown in Table 1.

### 2.8. Real-Time Polymerase Chain Reaction

Tissues were homogenized individually and dissolved in Trizol (Invitrogen, Carlsbad, CA, USA) to extract RNA. BALF was centrifuged at 1800 rpm and the supernatants were harvested and centrifuged at 4000 rpm; the methanolysis method was used to isolate RNA. The concentration of RNA was measured by NanoDrop 2000 spectrophotometer (Thermo Fisher, USA) and reverse transcribed with a RT Reagent Kit (Novoprotein, China). Amplification of cDNA was conducted in a DNA thermal cycler (Biometra, Germany). SYBR qPCR Supermix Plus (Novoprotein, China), primers, and cDNA were added in 8 straight tubes in StepOnePlus instruments (Thermo Fisher) to get the corresponding results with StepOnePlus™ Software v2.3. Primer sequences are listed in Table 2.

### 2.9. Gram Staining

The bacterial liquid was smeared in the middle of the slide. After natural drying at room temperature, fixing the bacteria on the slide via flame and staining in carbonate complex red dye solution were done. The slide was washed with water for 30 mins and being decolorized with hydrochloric acid alcohol. Finally, redyeing the slide with meilan was performed.

### 2.10. Statistical Analysis

All statistical tests were performed with GraphPad Prism 5 (GraphPad Software Inc., San Diego, USA). Significant differences between data sets were performed with either the one-way ANOVA for more than two groups or the two-way ANOVA for two variables or Tukey's multiple comparisons test (GraphPad Software Inc., San Diego, CA, USA). Data were represented as mean ± SD. ^∗∗∗^*P* < 0.001, ^∗∗^*P* < 0.01, ^∗^*P* < 0.05, and *P* > 0.05 show no significance, as stated in figure legends.

## 3. Results

### 3.1. The Percentages of CD3^+^, CD4^+^, and CD8^+^ T Cells in the Lavage Fluids, Nasal Mucosa, Trachea, Lungs, and Blood

**Figure 2:**
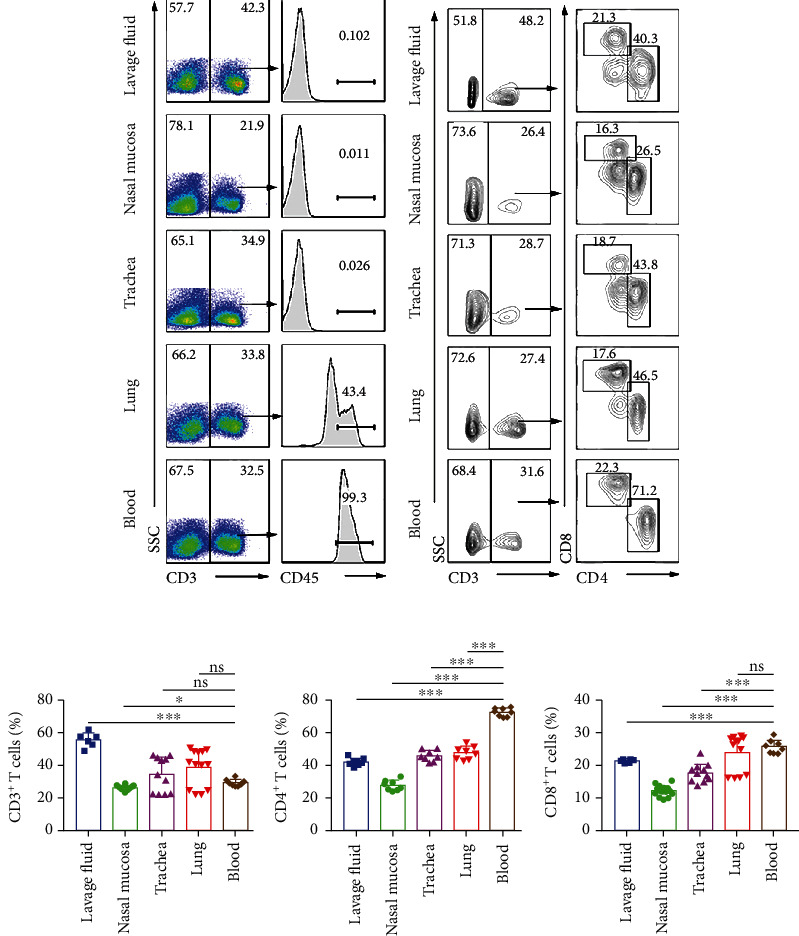
The proportions of CD3^+^, CD4^+^, and CD8^+^ T cells in the lavage fluids, nasal mucosa, trachea, lungs, and blood. CD45^−^CD3^+^ T cells (noncirculating T cells) and CD45^+^CD3^+^ T cells (circulating T cells) from lavage fluid, nasal mucosa, trachea, and lung tissues were distinguished by tail vein injection with fluorochrome-conjugated CD45 antibody. (a) Live and singlet CD3 lymphocytes from nasal mucosa, trachea, lung tissues and blood were gated and subsequently analyzed on the percentage of labeled CD45^+^ T cells by flow cytometry. (b) Live and singlet CD45^−^ lymphocytes from lavage fluid, nasal mucosa, trachea, and lung tissues and CD45^+^ lymphocytes from blood were gated and subsequently analyzed on CD3^+^, CD4^+^, and CD8^+^ T cells by flow cytometry. The tissue cells of the respiratory system were shown in the representative pseudocolor graphs. (c) The statistical results of noncirculating CD3^+^, CD4^+^, and CD8^+^ T cells in lavage fluids, nasal mucosa, trachea, and lungs and circulating CD3^+^, CD4^+^, and CD8^+^ T cells in blood were shown as mean ± SEM of eight independent experiments. Statistical significance was determined with one-way ANOVA for multiple comparisons. ^∗^*P* < 0.05 and ^∗∗∗^*P* < 0.001; ns: no significance.

To distinguish noncirculating T cells and circulating T cells in the respiratory tissues, we intravenously injected C57 mice with fluorochrome-conjugated CD45 antibody. As shown in Figure 2(a), 99.3% of T cells in peripheral blood were CD45^+^CD3^+^ cells, indicating that fluorescent antibodies against CD45 had been successfully labeled in mice. Meanwhile, CD45^−^CD3^+^ acyclic T cells in nasal mucosa and trachea accounted for more than 99%, but CD45^−^CD3^+^ acyclic T cells in the lungs accounted for 43.4%, and CD45^+^CD3^+^ acyclic T cells accounted for 56.6%, suggesting that we need intravenously injected mice with CD45Ab when we research lung tissue cells. The cells in the lavage fluids, nasal mucosa, trachea, lungs, and blood were stained with anti-CD3, anit-CD4, and anti-CD8 mAbs; live and singlet CD45^−^ lymphocytes from nasal mucosa, trachea, lung tissues and CD45^+^ lymphocytes from blood were gated and subsequently analyzed on CD3^+^, CD4^+^, and CD8^+^ T cells by flow cytometry (Figure 2(b)). The numbers of CD3^+^ T cells in lavage fluids were higher than those in other organs. Further analysis and comparison showed that the numbers of CD4^+^ T cells in blood were higher than those in others. The numbers of CD8^+^ T cells in blood were higher than those of lavage fluids, nasal mucosa, and trachea, but there is no difference in the lungs (Figure 2(c)).

### 3.2. Memory T Cells and Tissue-Resident Memory T Cells including CD4^+^ and CD8^+^ T Cells in the Lavage Fluids, Nasal Mucosa, Trachea, and Lungs

**Figure 3:**
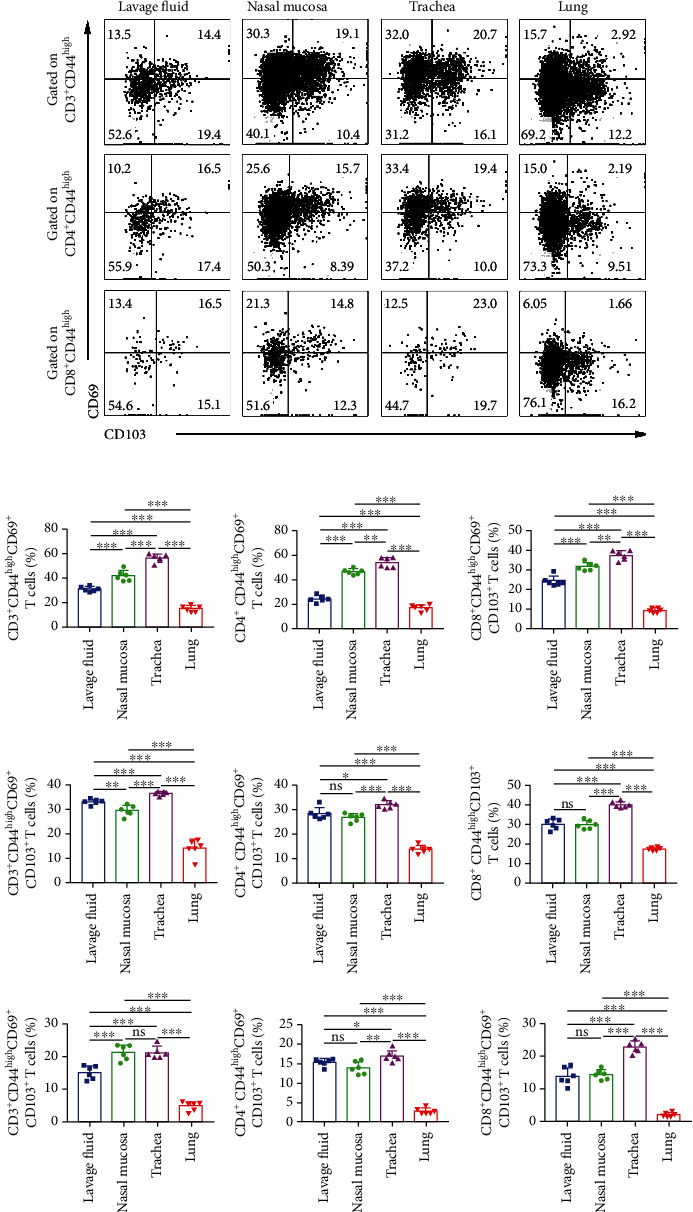
The tissue-resident memory T cells including CD4^+^ and CD8^+^ T cells in the lavage fluids, nasal mucosa, trachea, and lungs. (a) Live and singlet T_RM_ cells from lavage fluid, nasal, trachea, and lung tissues were gated on CD3^+^CD44^high^, CD4^+^CD44^high^, and CD8^+^CD44^high^ T cells and subsequently analyzed by flow cytometry. (b) The statistical results of CD69^+^ T, CD103^+^ T, and CD69^+^CD103^+^ T cells on CD3^+^CD44^high^, CD4^+^CD44^high^, and CD8^+^CD44^high^ T cells in the lavage fluids, nasal mucosa, trachea, and lungs were shown as mean ± SEM of six independent experiments. Statistical significance was determined with Tukey's multiple comparisons test. ^∗^*P* < 0.05, ^∗^*P* < 0.01, and ^∗∗∗^*P* < 0.001; ns: no significance.

To examine whether the proportions of T_RM_ cells in different respiratory tissues were the same, the lavage fluid, nasal mucosa, trachea, and lung cells from mice which were intravenously injected with fluorochrome-conjugated CD45 antibody were stained with anti-CD3, anit-CD4, anti-CD8, anti-CD44, anti-CD69, and anti-CD103 mAbs and gated on CD45^−^CD3^+^CD44^high^, CD45^−^CD4^+^CD44^high^, and CD45^−^CD8^+^CD44^high^, respectively, to analyze the percentages of CD69^+^ T, CD103^+^ T, and CD69^+^CD103^+^ T cells. In addition, we found that the proportion of memory T cells in bronchial lymph nodes (BLN) was significantly lower than that in peripheral blood but higher than that in nasal mucosa and tracheal tissue and the phenotype of memory T cells in BLN was similar to that in peripheral blood and respiratory tissue. (Supplementary Figures 1A–1B). The representative dot graphs of lavage fluid, nasal mucosa, trachea, and lung cells were shown (Figure 3(a)). We found that the percentages of CD69^+^ T cells and CD103^+^ T in memory T cells in the trachea are both higher than those from other organs, and those in the lungs were significantly lower than those in nasal mucosa and lavage fluid. In addition, the frequency of CD69^+^CD103^+^ T in CD4^+^CD44^high^ and CD8^+^CD44^high^ in the trachea was higher than that from other organs, but there is no significant difference with the nasal mucosa in CD3^+^CD44^high^. The proportion of CD69^+^CD103^+^ T in memory T cells in the lavage fluid, nasal mucosa, and trachea are significantly higher than that in the lungs (Figure 3(b)), thereby suggesting that the ratio of T_RM_ cells in the mucosa is higher than that of other tissues. It also suggests that intranasal immunization should be used to prevent respiratory diseases in modeling.

### 3.3. The Chemokine Receptors CCR5 and CXCR6 and Chemokine CXCL16 Are Highly Expressed on Memory T Cells in the Lavage Fluid, Nasal Mucosa, Trachea, and Lung Tissues

**Figure 4:**
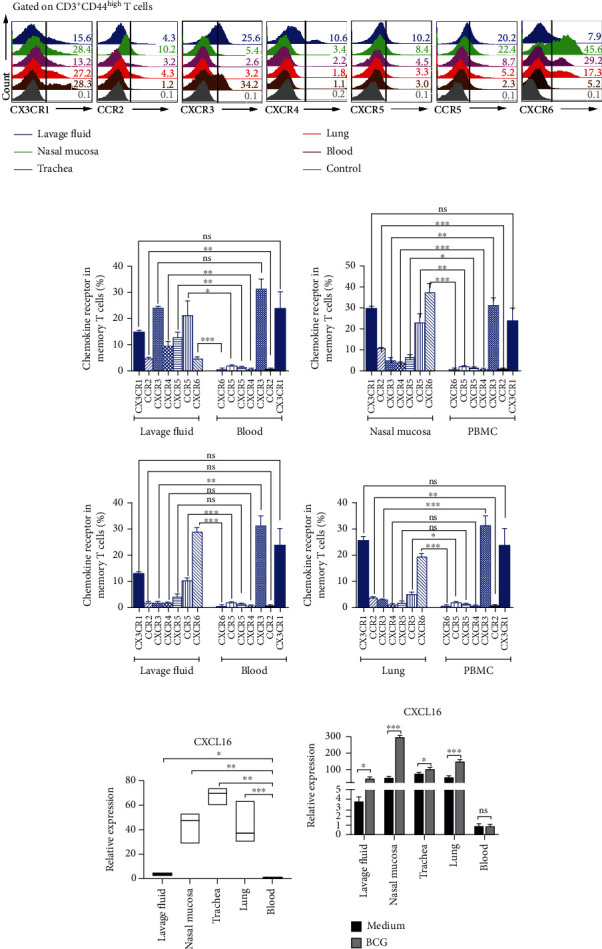
The expression of chemokine receptors between noncirculating T cells and circulating T cells. (a) The frequencies of CX3CR1, CCR2, CXCR3, CXCR4, CXCR5, CCR5, CXCR6, and CXCL16 on noncirculating T cells in nasal mucosa, trachea, and lung tissues and circulating T cells in blood were detected by flow cytometry and shown. (b) The statistical results of the frequencies of different chemokine receptors were analyzed by two-way ANOVA for multiple comparisons. (c) The statistical results of the expression of CXCL16 in the lavage fluid, nasal mucosa, trachea, lungs, and blood were analyzed by one-way ANOVA for multiple comparisons. (d) After intranasal vaccination with BCG, the statistical results of the expression of CXCL16 in the lavage fluid, nasal mucosa, trachea, lungs, and blood were analyzed by two-way ANOVA for multiple comparisons. ^∗^*P* < 0.05, ^∗∗^*P* < 0.01, and ^∗∗^*P* < 0.001; ns: no significance.

To further determine the different expression of chemokine receptors and chemokine, based on the results of flow cytometry, we found that there were significant differences in the chemokine receptors in acyclic T cells in the lavage fluid, nasal mucosa, trachea, and lungs and circulating T cells in blood. As shown in Figures 4(a) and 4(b), we detected 7 chemokine receptors and found that CCR5 and CXCR6 were significantly upregulated in lavage fluid, nasal mucosa, trachea, and lung tissues when compared with those in blood. In order to verify these results, we detected the expression of CXCL16, the ligand of CXCR6, in the lavage fluid, nasal mucosa, trachea, and lung tissues from noncirculating T cells and peripheral blood circulating T cells by flow cytometry. According to the results, lavage fluid, nasal mucosa, trachea, and lung tissues significantly increased the expression of CXCL16 compared with blood (Figure 4(c)). Furthermore, CXCL16 continued to be upregulated after intranasal vaccination with BCG (Figure 4(d)). Additionally, flow cytometry confirmed that the memory T cells that reside in tissues do not express CD62L and CCR7 (Supplementary Figure 1C); T cells in BALT and NALT did not coexpress CD62L and CCR6 but they expressed only a small amount of CD62L and CCR6 (Supplementary Figure 1D).

### 3.4. CD69^+^ and CD103^–^ T Cells Express Higher IFN-*γ* than CD69^–^ and CD103^+^ T Cells

**Figure 5:**
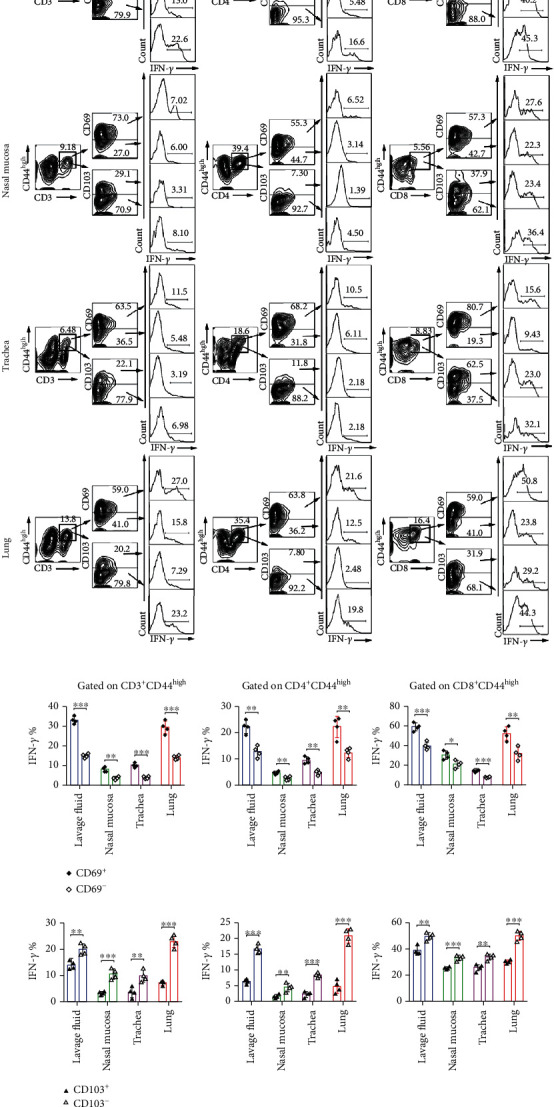
The expression of IFN-*γ* was higher in CD69^+^ and CD103^−^ T cells than CD69^−^ and CD103^+^ T cells when stimulated by PMA and ionomycin. (a) Live and singlet T_RM_ cells from lavage fluid, nasal, trachea, and lung tissues were gated on CD3^+^CD44^high^, CD4^+^CD44^high^, and CD8^+^CD44^high^ T cells and subsequently analyzed on the expression of IFN-*γ* by flow cytometry. All cells were stimulated for 6 hrs with PMA and ionomycin in the presence of BFA in flow tubes. (b) The cells were detected for the frequency of INF-*γ* in CD69^−/+^ or CD103^−/+^ cells by FACS. Statistical significance was determined with two-way ANOVA. ^∗^*P* < 0.05, ^∗∗^*P* < 0.01, and ^∗∗∗^*P* < 0.001.

To explore the functional differences of different phenotypes of T_RM_ cells from lavage fluids, nasal mucosa, trachea, and lung tissues, we separated those cells and stimulated for 6 hrs with PMA and ionomycin in the presence of BFA. Subsequently, we gated on CD3^+^CD44^high^, CD4^+^CD44^high^, and CD8^+^CD44^high^ T cells and analyzed on the frequency of expression of IFN-*γ* in CD69^−/+^ and CD103^−/+^ T cells by flow cytometry (Figure 5(a)), the results showed that no matter in CD3^+^CD44^high^, CD4^+^CD44^high^, and CD8^+^CD44^high^ T cells, CD69^+^ and CD103^–^ T cells expressed higher IFN-*γ* than CD69^–^ and CD103^+^ T cells (Figure 5(b)).

### 3.5. Vaccination of Mice with BCG Induced the Production and Expression of IFN-*γ*, IL-2, and TNF-*α* by CD3^+^, CD4^+^, and CD8^+^ T Cells in the Lavage Fluids, Nasal Mucosa, Trachea, and Lungs

C57BL/6 mice were intranasally (I.N.) immunized following the prime-boost protocol and challenged intranasally with BCG or PBS as a control once a week for 4 weeks (Figure 1(a)). Cells isolated from control and BCG group lavage fluid, nasal mucosa, trachea, lungs, and blood cells were suspended in complete RPMI 1640 medium at a final concentration of 2 × 10^6^ cells/ml. These cells were stimulated for 48 hrs with or without BCG plus anti-CD28 in a 96-well round plate. The production of IFN-*γ* was detected by ELISA. The results showed the cells from lavage fluid, nasal mucosa, trachea, and lung cells in the BCG group and stimulated with BCG in vitro producing higher levels of IFN-*γ*, but blood cells had no significance after the stimulation (Figure 1(b)). At the same time, we detected IFN-*γ* in peripheral blood plasma, BLF, and BALF of two groups of mice by ELISA. The results were shown in Figure 1(c); IFN-*γ* in NLF and BALF of immunized mice was significantly higher than that in the control group, but there was no significant difference in peripheral blood (Figure 1(c)). In addition, the cells from two groups were stimulated for 12 hrs with or without BCG plus anti-CD28 in the presence of BFA. The expression of IFN-*γ*, IL-2, and TNF-*α* was detected by flow cytometry. The results showed that expression of IFN-*γ*, IL-2, and TNF-*α* by CD3^+^, CD4^+^, and CD8^+^ T cells in the lavage fluids, nasal mucosa, trachea, and lungs is significantly higher in the BCG group than the control group (Table 3). Those data indicated that vaccination with BCG induced the production and expression of IFN-*γ*, IL-2, and TNF-*α* by CD3^+^, CD4^+^, and CD8^+^ T cells in the lavage fluids, nasal mucosa, trachea, and lungs.

### 3.6. BCG Induced the Expression of IFN-*γ* by Tissue-Resident Memory T Cells in the Lavage Fluid, Nasal Mucosa, Trachea, and Lungs

To further confirm the production of antigen-specific cytokine IFN-*γ* by T_RM_ subgroup CD69^−/+^CD103^−/+^ in various tissues of the respiratory system, the cells from respiratory tissue cells were stimulated separately with BCG (50 *μ*g/ml) plus anti-CD28 in the presence of BFA for 12 hrs and stained with anti-CD3, anti-CD44, anti-CD69, anti-CD103, and anti-IFN-*γ* mAbs. Gated on CD3 T cells, percentages of antigen-specific IFN-*γ* in CD69^−/+^CD103^−/+^ cells between the control and BCG groups were analyzed. The results showed that BCG induced the expression of IFN-*γ* by tissue-resident memory T cells CD69^+^CD103^−/+^ and CD69^−^CD103^−^ in the lavage fluid, nasal mucosa, trachea, and lungs. Interestingly, we found no significant difference in the ability of CD69^−^CD103^+^ cells to produce cytokine IFN-*γ* before and after immunization (Table 4).

### 3.7. Tissue-Resident Memory T Cells Generated by BCG Protected the Infection of Live BCG

**Figure 6:**
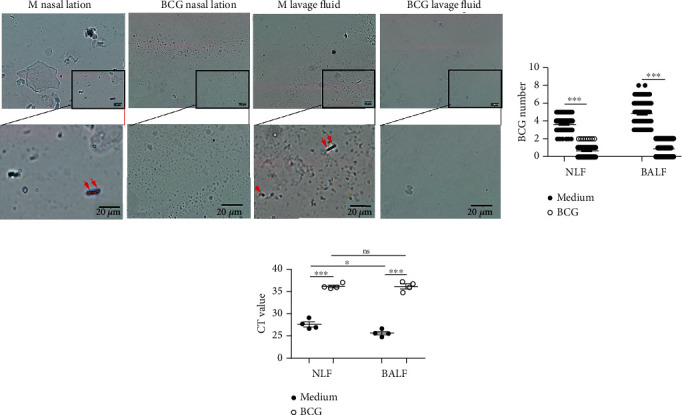
Antigen-specific tissue-resident memory T cells generated by BCG vaccination protected against the infection of live BCG. (a) NLF and BALF from mice after intranasal vaccination with or without BCG were infected intranasally with live BCG and stained with Gram (scale bar = 100 micros; magnification, 40x and 100x). (b) (*n* = 30) The statistical results of the numbers of BCG in NLF and BALF were counted in the slice of Gram staining vision; (c) (*n* = 4) The levels of BCG mRNA in nasal lavage fluid and lavage fluid were determined by qPCR as mean ± SEM. Statistical significance was determined with two-way ANOVA for multiple comparisons. ^∗^*P* < 0.05 and ^∗∗∗^*P* < 0.001; ns: no significance.

After intranasal vaccination of mice with BCG for 3 months, mice were infected intranasally with live BCG and sacrificed two days later. The results showed that the numbers of BCG in bronchoalveolar lavage fluid (BALF) and nasal lavage fluid (NLF) from control mice were much higher than BCG from vaccinated mice (Figures 6(a) and 6(b)). At the same time, the mRNA of BCG in control mice was also higher than that of BCG-vaccinated mice (Figure 6(c)). Those data indicated that BCG-vaccinated mice cleared up live BCG much faster than nonvaccinated mice in the respiratory system and had the ability to provide protection against pulmonary reinfection.

### 3.8. BCG-Specific Tissue-Resident Memory T Cells Persisted for a Long Period of Time in the Lavage Fluids, Nasal Mucosa, Trachea, and Lungs

**Figure 7:**
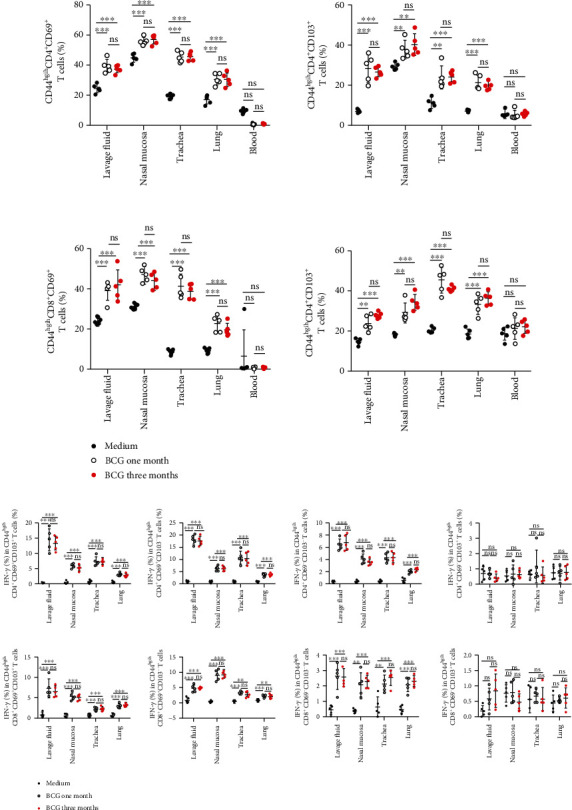
BCG-induced tissue-resident memory T cells persisted for a long period of time in the lavage fluids, nasal mucosa, trachea, and lungs. (a, b) (*n* = 5) After intranasal vaccination of mice with BCG for one and three months, the proportion of CD44^high^CD4^+^CD69^+^, CD44^high^CD4^+^CD103^+^, CD44^high^CD8^+^CD69^+^, and CD44^high^CD8^+^CD103^+^ T cells and the expression of antigen specific IFN-*γ* in the four groups of CD69^−/+^CD103^−/+^ T cells on CD4^+^ and CD8^+^ T cells were analyzed as mean ± SEM. The results representing four independent experiments for five mice each group were shown and compared with two-way ANOVA. ^∗∗^*P* < 0.01 and ^∗∗∗^*P* < 0.001; ns: no significance.

To explore the ratio and survival time of T_RM_ cells in the respiratory system, the cells from lavage fluid, nasal mucosa, trachea, and lungs were stained and analyzed for the proportion of CD44^high^CD4^+^CD69^+^, CD44^high^CD4^+^CD103^+^, CD44^high^CD8^+^CD69^+^, and CD44^high^CD8^+^CD103^+^ T cells after intranasal vaccination of mice with BCG for a month and three months. The results showed that T_RM_ cells were higher in vaccinated group than the control group which did not decline with time (Figure 7(a), *n* = 5). The expression of antigen-specific IFN-*γ* in CD69^−/+^CD103^−/+^ subset T cells from CD4^+^ and CD8^+^ T cells (Figure 7(b)) showed that expression of IFN-*γ* in different subgroup T_RM_ cells was increased significant in the lavage fluid, nasal mucosa, trachea, and lungs of BCG-vaccinated mice.

## 4. Discussion

In the current study, intranasal vaccination of mice with BCG was applied to investigate the role of T_RM_ cells in the prevention of respiratory infection [20, 21]. Our study found that a higher proportion of T_RM_ cells was enriched in nasal mucosa, which further suggested that intranasal vaccination was more suitable for the animal models of this study. Our results showed that intranasal vaccination of mice with BCG protects the mouse respiratory system from BCG infection by promoting the proportion of T_RM_ cells and release of antigen-specific cytokines of IFN-*γ*.

T_RM_ cells are noncirculating lymphocytes, which are distributed in nonlymphoid tissues including the skin and mucosal tissues, the urogenital system, brain tissue, the respiratory system, the liver, and other tissues. When pathogenic microorganisms invade, they can directly and rapidly produce a defense response and play a very important role. However, there are few comparative studies in respiratory tissues such as the nasal mucosa, trachea, lungs, and lavage fluid. Earlier studies reported that T_RM_ cells were highly activated and remained in the lungs long after the respiratory virus infection that had been cleared which is associated with high CD69 expression [22, 23]. However, T_RM_ cells have recently been reported to maintain homeostasis and inhibit bacterial infection in the respiratory system [2, 24]. Mueller and Mackay found that the adoptive transfer experiments showed that lung tissue-resident CD4^+^ T cells homed back to the lungs and help to mediate bacterial clearance [9]. Furthermore, airway CD8^+^ tissue-resident memory T cells can quickly respond to limit early viral replication following secondary influenza virus challenge [25]. And the maintenance of tissue homeostasis is critically dependent on the function of tissue-resident immune cells [9]. Most of these investigations of T_RM_ cells were focused on the lungs, skin, etc. Recently, our team proposed that intranasal vaccination offer the best protection against respiratory infection in mice [26]. This work encourages us to further explore the effects of nasal immunization on the proportion and function of T_RM_ cells in various respiratory tissues.

T_RM_ cells are tissue-resident cells characterized by expression of high CD69 and/or CD103, which play an important role in the maintenance of respiratory homeostasis and autoimmune diseases [27, 28]. In the current studies, we found that in the BCG model, the proportion of T_RM_ cells increased in the lavage fluid, nasal mucosa, trachea, and lungs. Furthermore, T_RM_ cells stimulated by BCG in vitro can produce the antigen-specific cytokine IFN-*γ*. Emerging evidence suggests that CXCR6 regulates localization of tissue-resident memory CD8 T cells to the airways [29], which is consistent with the results of our study. Our results also confirmed that the expression of CXCR6 on the surface of noncirculating memory T cells in lavage fluid, nasal mucosa, trachea, and lung tissues was higher than that of circulating T cells in peripheral blood, so it was speculated that CXCR6 was related to the residence of noncirculating T cells in respiratory tissues. Further RT-PCR detection revealed that the mRNA level of only CXCR6 chemokine CXCL16 in lavage fluid, nasal mucosa, trachea, and lung tissues was significantly higher than that in peripheral blood. These results suggested that the maintenance of noncirculating T cells in respiratory tissues was probably related to CXCR6-CXCL16. Besides, we found that BCG survived less in the upper and lower respiratory tract lavage fluid in the BCG model of intranasal vaccination after being attacked by BCG live bacteria compared to control mice. Thus, our study demonstrated that the ratio of T_RM_ in the nasal mucosa is higher than other respiratory tissues. BCG induced the expression of IFN-*γ* by T_RM_ cells in lavage fluid, nasal, trachea, and lung tissues. T_RM_ cells are generated by BCG and protected the infection of live BCG. In addition, intraepithelial lymphocytes (IELs) expressing *γδ*T cell receptors (TCRs) have been shown to be tissue-resident T cells that play a key role in immune surveillance [30]. Unfortunately, we failed to differentiate intraepithelial lymphocytes (IEL) and non-IELS. We believe that IELs may induce a complex immune response involving being defensive in reinfection. Therefore, future studies with comprehensive profiling of IELS and non-IEL in various tissues of the respiratory system are necessary to identify their functional differences.

T_RM_ cells were increased in the respiratory system after intranasal vaccination of mice with BCG. Meanwhile, consistent with reports [31] that BCG-specific T_RM_ cells persisted for a long time in lavage fluid, nasal, trachea, and lung tissues, our study suggests that intranasal vaccination may be the best way to induce the immune responses by activating T_RM_ cells in the mucosal tissues of the respiratory system and preventing the diseases in the respiratory system.

## 5. Conclusions

Our results indicate that activation and establishment of T_RM_ cells in the respiratory system may have an important role in providing protection against pulmonary reinfection, and subsequently trigger defense response directly and quickly when the pathogens invade in the tissues. In conclusion, our results elucidate the role of the T_RM_ cells in the disease of the respiratory system.

## Figures and Tables

**Table 1:** The antibodies used in flow cytometry.

Antibody name	Supplier	Fluorochrome
CD3	BD	PE-CF594/PE
CD4	BD	APC-cy7/FITC
CD8	BD	percp-cy5.5/AF700
CD44	BD	APC-cy7/percp-cy5.5
CD69	BD	PE
CD103	BioLegend	PE-cy7
IFN-*γ*	BD	APC
TNF-*α*	BD	PE
IL-2	BioLegend	PE

**Table 2:** Sequence of primers used for RT-PCR.

Gene	5′ to 3′
EspC	F: CACTGCGGGCAGCAAACGTGG
	R: TAAACGGAAGGGACACGATCA

CXCL16	F: GCAGGGTACTTTGGATCACATCC
	R: AGTTCACGGACCCACTGGTCTT

GAPDH	F: TCAATGAAGGGGTCGTTGAT
	R: CGTCCCGTAHACAAAATGGT

**Table 3:** The expression of cytokines in T cells of the respiratory system and in blood before and after immunization.

Name	Sample	Medium group (mean %)	BCG group (mean %)	*P* value
IFN-*γ* in T cells	Lavage fluid	0.365	1.8375	0.0004
	Nasal mucosa	0.45	3.18	0.0001
	Trachea	0.34	2.2425	0.0003
	Lung	0.2225	2.8375	0.00009
	Blood	0.3375	0.37	0.5643

IL-2 in T cells	Lavage fluid	1.2133	6.1233	0.042
	Nasal mucosa	0.6967	5.41	0.014
	Trachea	0.58	4.7433	0.0054
	Lung	0.47	4.66	0.0034
	Blood	0.7967	0.75	0.9062

TNF-*α* in T cells	Lavage fluid	0.5633	5.3033	0.0009
	Nasal mucosa	0.8033	9.0633	0.0206
	Trachea	1.2167	5.8367	0.0052
	Lung	0.7067	4.4767	0.004
	Blood	1.4067	0.9833	0.5419

IFN-*γ* in CD4^+^ T cells	Lavage fluid	1.0075	4.2375	0.0004
	Nasal mucosa	1.165	4.055	0.0043
	Trachea	0.715	4.46	0.0018
	Lung	0.8175	2.415	0.0104
	Blood	0.5025	0.625	0.5091

IL-2 in CD4^+^ T cells	Lavage fluid	0.7967	3.7633	0.0008
	Nasal mucosa	0.6133	4.9633	0.0018
	Trachea	0.4	6.2733	0.0007
	Lung	0.4833	4.21	0.0025
	Blood	0.5767	0.6333	0.8156

TNF-*α* in CD4^+^ T cells	Lavage fluid	1.02	4.7367	0.0121
	Nasal mucosa	0.8867	9.6867	0.0008
	Trachea	1.8867	4.93	0.0187
	Lung	0.57	6.3867	0.0005
	Blood	0.73	0.8267	0.7998

IFN-*γ* in CD8^+^ T cells	Lavage fluid	0.475	2.72	0.0004
	Nasal mucosa	0.6025	2.1175	0.005
	Trachea	0.35	2.775	0.0008
	Lung	0.485	2.2675	0.0029
	Blood	0.1825	0.205	0.5528

IL-2 in CD8^+^ T cells	Lavage fluid	1.0433	6.63	0.0002
	Nasal mucosa	1.1467	6.7067	0.0012
	Trachea	0.6733	7.2567	0.0004
	Lung	0.8567	5.6567	0.0020
	Blood	1.06	0.6367	0.0505

TNF-*α* in CD8^+^ T cells	Lavage fluid	0.7867	6.0333	0.004
	Nasal mucosa	0.7467	8.8333	0.0015
	Trachea	0.7567	7.0033	0.0011
	Lung	0.3537	4.79	0.0016
	Blood	1.3467	0.84	0.2428

Vaccination of mice with BCG induced the production and expression of IFN-*γ*, IL-2, and TNF-*α* by T cells in the lavage fluids, nasal mucosa, trachea, and lungs. Respiratory system tissues of lavage fluid, nasal mucosa, trachea, lung, and blood cells were stimulated for 12 hrs with or without BCG plus anti-CD28 in the presence of BFA in flow tubes. The cells were detected for the frequencies of INF-*γ*, IL-2, and TNF-*α* by FACS as mean. Statistical significance was determined with two-way ANOVA. ^∗^*P* < 0.05, ^∗∗^*P* < 0.01, and ^∗∗∗^*P* < 0.001; *P* > 0.05: no significance.

**Table 4:** The expression of IFN-*γ* in T_RM_ subsets of respiratory tissues before and after immunization.

Subset of T_RM_ cells	Sample	Medium group (mean %)	BCG group (mean %)	*P* value
IFN-*γ* in CD69^+^CD103^+^ T cells	Lavage fluid	0.1825	5.055	0.0003
	Nasal mucosa	0.6025	2.71	0.0033
	Trachea	0.6	5.23	0.00008
	Lung	0.4325	2.705	0.0001

IFN-*γ* in CD69^+^CD103^−^T cells	Lavage fluid	0.2925	9.5875	0.0002
	Nasal mucosa	0.3365	4.405	0.00009
	Trachea	0.695	8.55	0.00007
	Lung	0.6775	4.27	0.00008

IFN-*γ* in CD69^−^CD103^−^T cells	Lavage fluid	0.415	3.625	0.0005
	Nasal mucosa	0.3025	2.125	0.0021
	Trachea	0.545	2.1725	0.001
	Lung	0.595	1.79	0.0017

IFN-*γ* in CD69^−^CD103^+^T cells	Lavage fluid	0.21	0.4875	0.104
	Nasal mucosa	0.545	0.4425	0.526
	Trachea	0.6625	0.7725	0.6259
	Lung	0.515	0.8275	0.2016

BCG induced the expression of IFN-*γ* by tissue-resident memory T cells in the lavage fluids, nasal mucosa, trachea, and lungs. Respiratory system tissues of lavage fluid, nasal mucosa, trachea, lung, and blood cells were stimulated for 12 hrs with or without BCG plus anti-CD28 in the presence of BFA in flow tubes. The expression of antigen specific IFN-*γ* in the four groups of CD69^-/+^CD103^-/+^ T cells on CD44^high^CD3^+^ T cells were analyzed. The statistical results of the expression of antigen-specific IFN-*γ* in the four groups of CD69^−/+^CD103^−/+^ T cells as mean. The significance was compared with two-way ANOVA. ^∗∗^*P* < 0.01 and ^∗∗∗^*P* < 0.001; *P* > 0.05: no significance.

## Data Availability

The (original) data used to support the findings of this study are included within the article.

## Supplementary Materials

Supplementary Materials Supplementary Figure 1: (A-B) comparison of memory T cells in bronchial lymph nodes and other tissues; (C) expression of L-selectin or CCR7 by memory T cells in all tissues; (D) expression of CCR6 and L-selectin by T cells in BALT and NALT. Statistical significance was determined with one-way ANOVA. ^∗^*P* < 0.05 and ^∗∗^*P* < 0.01; ns: no significance.
