# Supplementary figures and images for: Antigen-Specific Tissue-Resident Memory T Cells in the Respiratory System Were Generated following Intranasal Vaccination of Mice with BCG
Source: J Immunol Res. 2021 Mar 27;2021:6660379. doi: 10.1155/2021/6660379 (PMC8019380; doi:10.1155/2021/6660379)

**A**Gated on CD3<sup>+</sup> T cells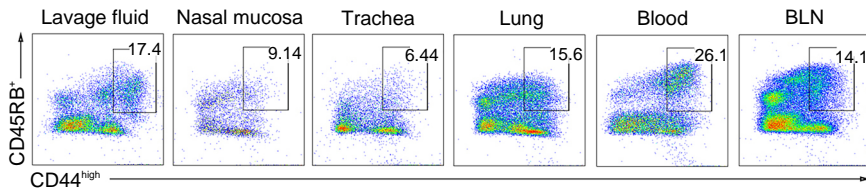**B**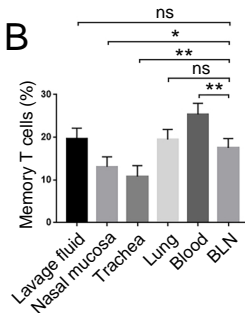**C**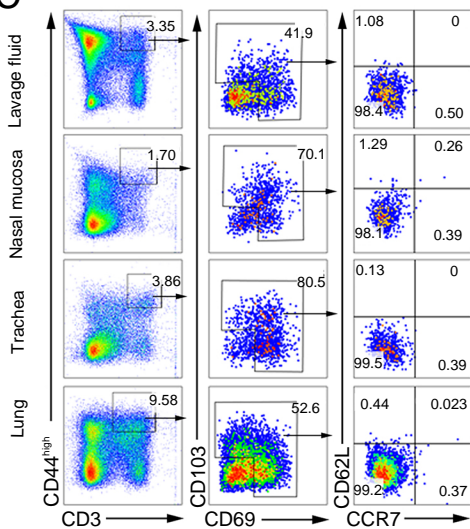**D**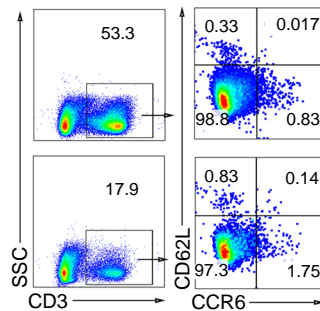

Supplement: Supplementary Materials — Supplementary Figure 1: (A-B) comparison of memory T cells in bronchial lymph nodes and other tissues; (C) expression of L-selectin or CCR7 by memory T cells in all tissues; (D) expression of CCR6 and L-selectin by T cells in BALT and NALT. Statistical significance was determined with one-way ANOVA. ∗P < 0.05 and ∗∗P < 0.01; ns: no significance. [file 6660379.f1.pdf]
